# Hydrostatic pressure induces profibrotic properties in hepatic stellate cells via the RhoA/ROCK signaling pathway

**DOI:** 10.1002/2211-5463.13405

**Published:** 2022-04-15

**Authors:** Zisheng Huang, Mahmoud Osman Khalifa, Weili Gu, Tao‐Sheng Li

**Affiliations:** ^1^ Department of Stem Cell Biology Atomic Bomb Disease Institute Nagasaki University Japan; ^2^ Department of Stem Cell Biology Nagasaki University Graduate School of Biomedical Sciences Japan; ^3^ Department of Hepatopancreatobiliary Surgery Guangzhou First People's Hospital China

**Keywords:** hepatic stellate cells, hydrostatic pressure, liver fibrosis, mechanotransduction, RhoA/ROCK

## Abstract

Elevated interstitial fluid hydrostatic pressure is commonly observed in diseased livers. We herein examined the hypothesis that hydrostatic pressure induces hepatic stellate cells to acquire profibrotic properties under pathological conditions. Human hepatic stellate cells were exposed to 50 mmHg pressure for 24 h. Although we observed few changes of cell growth and morphology, PCR array data on the expression of fibrosis‐associated genes suggested the acquisition of profibrotic properties. The exposure of hepatic stellate cells to 50 mmHg pressure for 24 h also significantly enhanced the expression of RhoA, ROCK1, α‐SMA, TGF‐β_1_, p‐MLC, and p‐Smad2, and this was effectively attenuated by the ROCK inhibitor Y‐27632. Our *ex vivo* experimental data suggest that elevated interstitial fluid hydrostatic pressure under pathological conditions may promote liver fibrosis by inducing acquisition of profibrotic properties of hepatic stellate cells through the RhoA/ROCK signaling pathway.

## Graphical abstract

In this study we demonstrated that elevated fluid hydrostatic pressure under pathological conditions may subsequently promote liver fibrosis by activating hepatic stellate cells through the RhoA/ROCK signaling pathway, providing a possible target for preventing fibrosis progression.

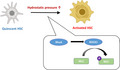

**Abbreviations**
DAPI: 4, 6‐diamidino‐2‐phenylindole
ECM: extracellular matrix
HSCs: hepatic stellate cells
MLC: myosin light chain
RhoA: ras homolog family member A
ROCK: rho‐associated protein kinase
RT‐qPCR: reverse transcription‐quantitative polymerase chain reaction

Fibrosis is the most common pathological manifestation of various chronic liver diseases, and the activation of hepatic stellate cells (HSCs) is already known to be the central event underlying liver fibrosis [1, 2]. Although the increase of inflammatory cytokines and the dysregulation of the extracellular matrix (ECM) have been demonstrated to induce the activation of HSCs [1, 2], the precise mechanisms on liver fibrosis have not yet been completely understood because of the complex changes of systemic and regional tissue microenvironments under various pathological conditions.

Biomechanical forces are well known to play essential roles in the development and homeostasis maintenance of our tissues/organs under physiological conditions [3]. Many mechanosensors, such as the mechanically sensitive ion channels and G‐protein‐coupled receptors on cell membranes have been demonstrated to sensitize the dynamics of biomechanical forces (i.e., mechanosensation), and then transduce into intracellular signaling pathways (i.e., mechanotransduction) to responsively modify the biological activities of cells [4, 5].

Alternations of biomechanical forces are generally observed in the liver under various pathological conditions [6, 7]. The inflammation‐induced fluid trapping in the acute phase will quickly elevate the interstitial fluid hydrostatic pressure, and the excessive deposition of ECM in the chronic phase results in the change of stiffness. For example, the hepatic venous pressure is known to be 7–12 mmHg in the healthy liver [8], but can be elevated to 30 mmHg in diseased livers [9]. The alterations of biomechanical forces have also been demonstrated to involve the initiation and progression of liver diseases, by changing the biological properties of HSCs, hepatocytes, and sinusoidal endothelial cells [10, 11]. Moreover, it has recently been reported that the elevation of interstitial fluid hydrostatic pressure in liver at the early onset of inflammation induces the phenotypic change of fibroblasts into myofibroblasts to synthesize ECM [12, 13]. Although the important roles of biomechanical forces in liver diseases have been highlighted in recent years [14], it is still suggested to further identify the molecular and cellular mechanisms.

The cytoskeleton is one of the prevailing ways for intracellular mechanotransduction [4]. Ras homolog family member A (RhoA), a key factor of cytoskeletal regulation and actin stress fiber formation, has been reported to highly expressed in fibrotic liver tissue [15]. Rho‐associated protein kinase (ROCK), as a downstream effector of RhoA, plays a critical role in inducing the phosphorylation of myosin light chain (MLC) to promote the formation of stress fibers [16]. Additionally, the ROCK inhibitor Y‐27632 has been demonstrated to attenuate the carbon tetrachloride‐induced liver fibrosis in rat by inhibiting the activation of HSCs [17]. Based on past studies, we hypothesize that elevated hydrostatic pressure induces the activation of HSCs via the RhoA/ROCK signaling pathway.

By using a commercial device to *ex vivo* mimic the elevated interstitial fluid hydrostatic pressure, we herein investigated the potential role and relevant mechanism of hydrostatic pressure in mediating the alternation of biological property of HSCs.

## Materials and methods

### Human HSCs

Primary human HSCs were purchased from ScienCell Research Laboratories (ScienCell, Carlsbad, CA, USA). Cells were expanded by using stellate cell medium (SteCM) (ScienCell), in a humidified incubator under 5% CO_2_ and 95% air at 37 °C. The third‐passaged cells were used for the following experiments.

### *Ex vivo* hydrostatic pressurization of human HSCs

A pneumatic pressurizing system (Strex, Osaka, Japan) was used to induce hydrostatic pressure. Briefly, HSCs were seeded in 60‐mm diameter culture dishes (5 × 10^4^ cells·dish^−1^), and incubated for 3 days until the cells formed around 70% confluence. The cell culture dishes were then randomly selected to move in a sealed chamber, in which the 20 or 50 mmHg pressure was stably induced by the pneumatic pressurizing system. Cell culture dishes without hydrostatic pressurization were used for the control.

### Cell morphology and viability

After 24 h of exposure to hydrostatic pressure, the morphology of HSCs was quickly observed under a microscope (Olympus IX71, Olympus, Tokyo, Japan). Then we harvested the HSCs from culture dishes using 0.25% trypsin. The number and viability of HSCs were analyzed by a TC20™ automated cell counter (Bio‐Rad, Hercules, CA, USA). The harvested cells were used for the analysis as the following.

### Human fibrosis RT2 Profiler™ PCR array

The RT2 Profiler™ PCR array was applied to screen for the genes related to fibrosis that probably had changes in expression, as previously described [18]. Briefly, total RNA was isolated from the cells using Quick‐RNA^TM^ MicroPrep Kit (Zymo Research, Irvine, CA, USA). RNA concentration and purity were measured by a NanoDrop 2000 spectrophotometer (Thermo‐Fisher Scientific, Waltham, MA, USA). A total of 500 ng RNA mixture equivalently collected from three independent experiments of each group was used to generate cDNA using the RT2 First Strand Kit (SABiosciences, Qiagen, Chatsworth, CA, USA). The human fibrosis RT2 Profiler™ PCR array was performed according to the manufacturer’s instructions. A total of 84 genes involved in fibrosis was included in the array. The fold change of expression was calculated using a web‐based data analysis program (SABiosciences).

### RT‐qPCR analysis

RT‐qPCR was performed to evaluate the expression of *RHOA*, *ROCK1*, *ROCK2*, *ACTA2*, and *TGFB1*. Briefly, HSCs were exposed to 50 mmHg pressure for 24 h as above, with or without 10 μm Y‐27632, a pan‐ROCK inhibitor (ATCC, Rockville, MD, USA) in medium. Total RNA was isolated from the HSCs using the Quick‐RNA^TM^ MicroPrep Kit as above, and 1.25 μg RNA was reverse‐transcribed using the SuperScript™ VILO™ cDNA Synthesis Kit (Thermo‐Fisher Scientific). Quantitative PCR was carried out with the SYBR Green real‐time PCR Master Mix (Toyobo, Osaka, Japan). The reactions were performed on a CFX96^TM^ real‐time PCR System (Bio‐Rad). The primer sequences are shown in Table S1. *GAPDH* was used for normalization.

### Western blot analysis

Cells were washed twice in PBS and solubilized in RIPA buffer for 30 min on ice. The lysates were clarified by centrifugation and the protein concentration was then determined using the Pierce™ BCA Protein Assay Kit (Thermo‐Scientific). A total of 20 μg of protein were loaded onto an SDS/polyacrylamide gels. The separated bands were transferred onto PVDF membranes. After blocking for 60 min in 5% nonfat milk, membranes were incubated with primary antibodies (Table S2) at 4 °C overnight. Membranes were washed and then incubated with secondary antibodies (Table S3) for 1 h at room temperature. The expression was visualized with SuperSignal™ West Femto Maximum Sensitivity Substrate (Thermo‐Scientific) and detected using ImageQuant LAS 4000 mini (GE Healthcare Life Sciences, Chicago, IL, USA). Semiquantification was done using image j software (NIH, Bethesda, MD, USA).

### Immunofluorescence analysis

Immunofluorescence analysis was also performed to detect the protein levels of RhoA, ROCK1, ROCK2, α‐SMA, TGF‐β_1_, p‐MLC, and p‐Smad2. Briefly, HSCs were seeded onto 4‐well culture chamber slides. When forming to around 70% confluence, the slides was exposed to 50 mmHg pressure for 24 h as above, with or without the addition of 10 μm Y‐27632 in medium. The cells were fixed by 4% paraformaldehyde at room temperature for 10 min, and then incubated in 0.5% Triton X‐100 at room temperature for another 10 min. After blocking by 10% bovine serum albumin at room temperature for 30 min, the cells were incubated with primary antibodies (Table S2) overnight at 4 °C, followed by incubation with a secondary antibody (Table S3) for 1 h at room temperature in the dark. F‐actin fibers were stained with TRITC‐phalloidin in mounting medium (Vectorlabs, Burlingame, CA, USA). Nuclei were stained with 4, 6‐diamidino‐2‐phenylindole (DAPI). Immunofluorescences were detected using an inverted fluorescence microscope (Olympus FV10i, Olympus). For each staining, at least 10 images were taken from randomly selected fields at 60× magnification, and the mean fluorescence intensity was measured by image j software (NIH).

### Statistical analysis

All the results are presented as the mean  ±  SD. Statistical significance was determined by one‐way ANOVA followed by Tukey’s test (Dr. SPSS II, Chicago, IL, USA). *P* <  0.05 was accepted as significant.

## Results

### The exposure of HSCs up to 50 mmHg hydrostatic pressure showed few changes in cell morphology and viability

**Fig. 1:**
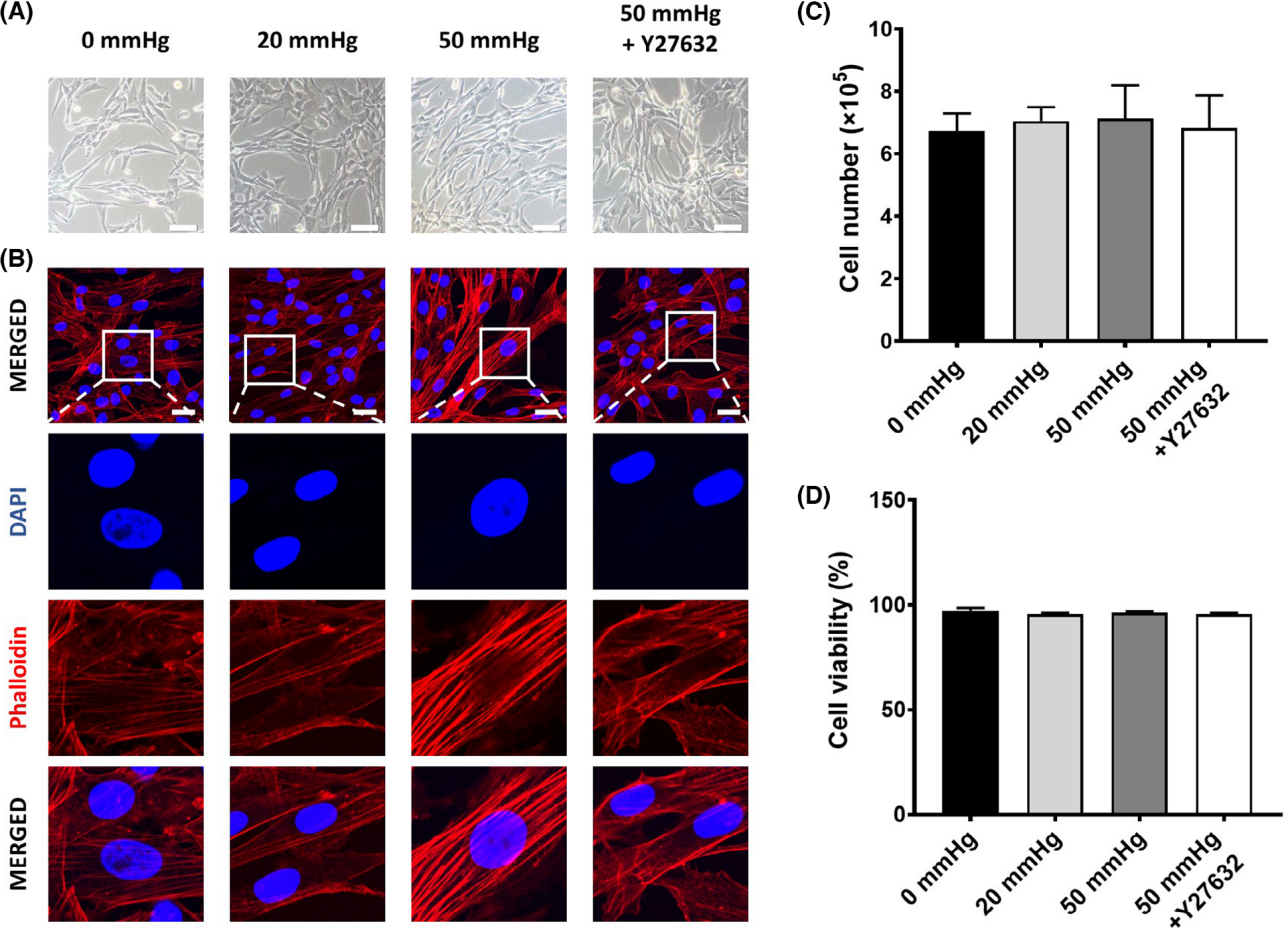
The morphology and cell viability of human hepatic stellate cells (HSCs) after exposure to 0, 20, or 50 mmHg pressure for 24 h with or without the addition of Y‐27632 in medium. (A) Representative phase‐contrast images show the morphology of HSCs under a light microscope. Scale bar = 200 μm. (B) Representative images of phalloidin staining shows the formation of F‐actin in HSCs. Scale bars = 30 μm. Quantitative data indicate the total cell count (C) and cell viability (D) from three independent experiments. Data are represented as mean ± SD.

As shown in the representative images (Fig. 1A), these HSCs displayed a typical morphology of a spindle‐shaped cell body and elongated nuclear. Compared to the control, the morphology of HSCs did not obviously change after exposure to 20 or 50 mmHg pressure for 24 h. Phalloidin staining for F‐actin revealed that HSCs exposed to 50 mmHg pressure formed a dense network of actin, with significantly thickened actin bundles (Fig. 1B). The formation of actin stress fibers in HSCs with 50 mmHg exposure were effectively blocked by Y‐27632 (Fig. 1B). The cell number and viability of HSCs were also comparable between the control treatment and hydrostatic pressure exposures (Fig. 1C,D).

### The exposure of HSCs to 50 mmHg hydrostatic pressure induced the acquisition of a profibrotic property

**Fig. 2:**
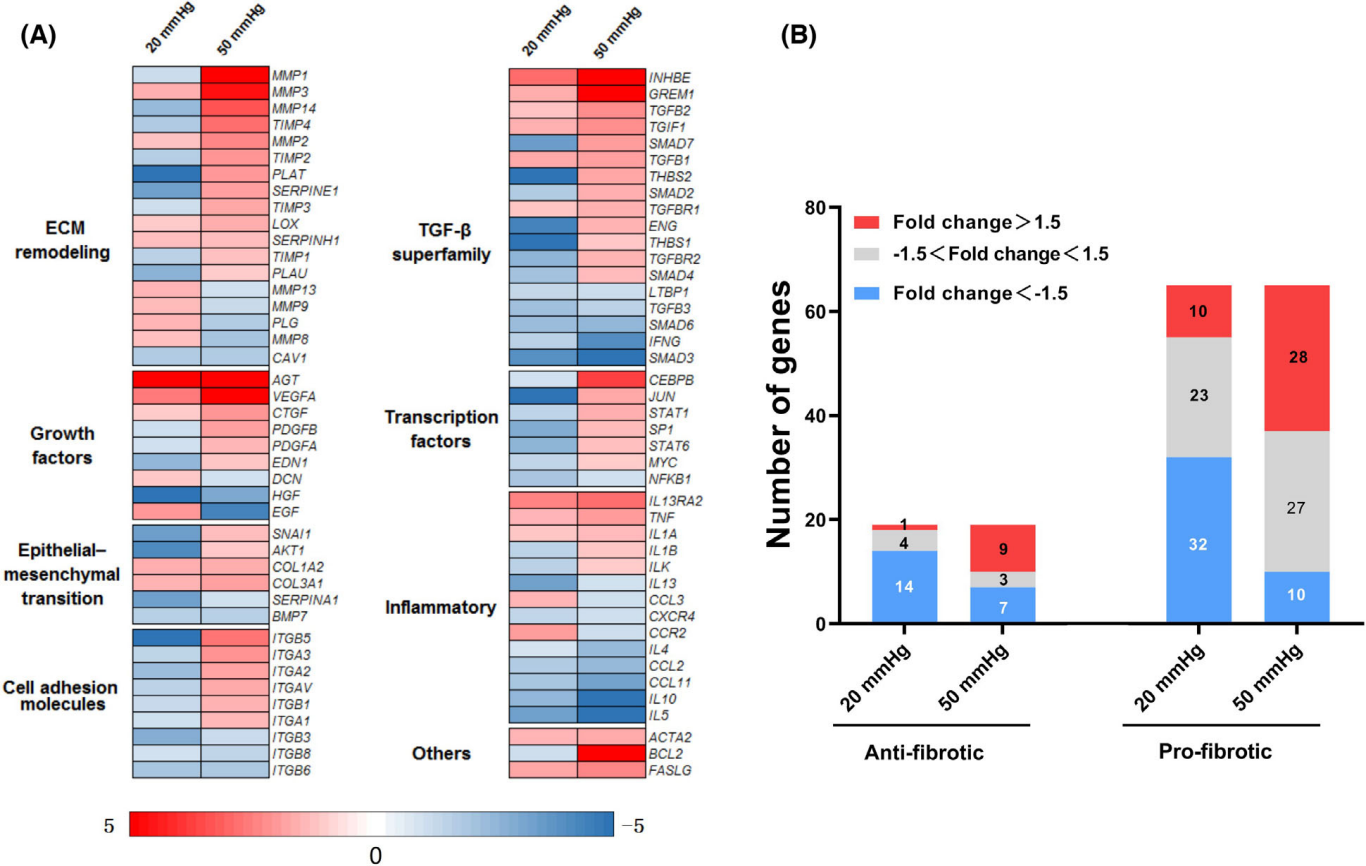
RT2 Profiler™ PCR array analysis on the expression of genes involved in fibrosis in human hepatic stellate cells (HSCs) after exposure to 0, 20, or 50 mmHg pressure for 24 h. (A) Heatmap depicts the expression changes (vs. 0 mmHg) of all genes belonging to different functional categories. (B) The numbers of up‐ and downregulated genes are counted according to the fold changes of expression and the biological functions (pro‐ or antifibrotic).

By pathway‐focused PCR array analysis, we widely investigated the expression of genes involved in fibrogenesis. Compared with the control treatment by 0 mmHg pressure, a large number of genes were up‐ or downregulated in HSCs with 24 h of exposure to 20 or 50 mmHg pressure (Table S4). According to the functional categories, we depicted the expression changes of genes into a heat map (Fig. 2A). Genes belonging to the TGF‐β superfamily, ECM remodeling, and cell adhesion molecules were extensively upregulated in HSCs after 24 h of exposure to 50 mmHg pressure. In contrast, gene expression was mildly changed after 24 h of exposure to 20 mmHg pressure. We could also confirm that the exposure to 50 mmHg pressure upregulated *ACTA2*, which encodes the protein of α‐SMA, a marker for the activated HSCs. The overall changes on the gene expression indicated the activation of HSCs exposed to 50 mmHg pressure.

Based on the general role on fibrogenesis, we further tried to roughly divide these genes into antifibrotic and profibrotic subgroups (Fig. 2B). We found that a larger part of profibrotic genes was upregulated more than 1.5‐fold in the HSCs exposed to 50 mmHg pressure (Fig. 2B), suggesting the acquisition of a profibrotic property.

### RhoA/ROCK signaling involved in the activation of HSCs with hydrostatic pressure exposure

**Fig. 3:**
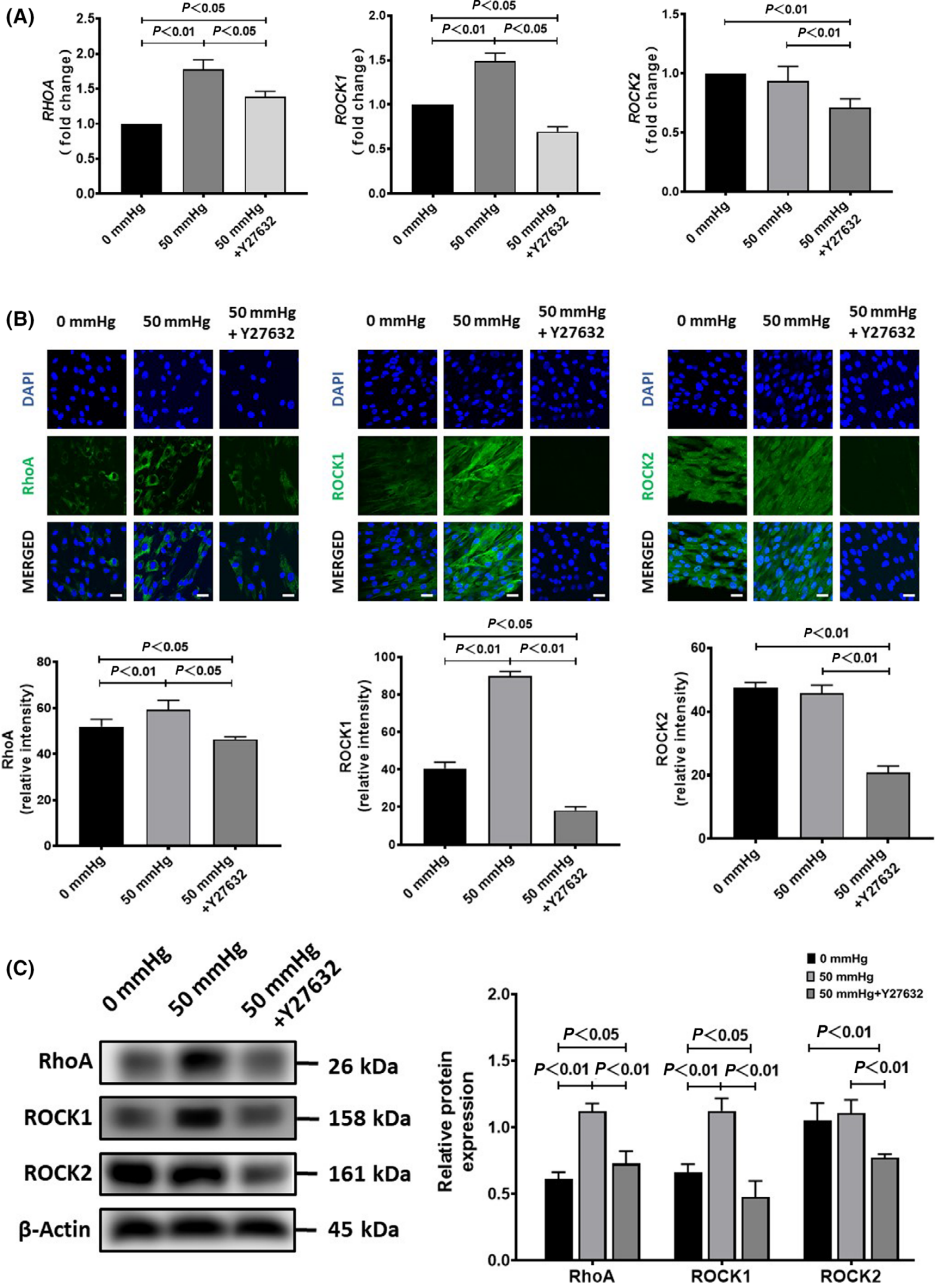
The expression of RhoA, ROCK1, and ROCK2 in human hepatic stellate cells (HSCs) after exposure to 50 mmHg pressure for 24 h with or without the addition of Y‐27632 in medium. (A) Quantitative RT‐qPCR data on the fold change of mRNA expression levels (vs. 0 mmHg). (B) Representative images (upper) and semiquantitative data on the immunofluorescence staining intensity. Scale bars = 30 μm. (C) The protein expression by western blot analysis is also shown. Data are represented as mean ± SD from three independent experiments. *P* values were analyzed by one‐way ANOVA.

To understand whether RhoA/ROCK signaling was involved in the activation of HSCs, we further evaluated the expressions of RhoA, ROCK1, and ROCK2 at the mRNA and protein levels. Data from RT‐qPCR analysis showed that the exposure to 50 mmHg pressure for 24 h significantly upregulated *RHOA* and *ROCK1*, but hardly changed *ROCK2* (Fig. 3A). Results of the immunofluorescence staining and western blot also confirmed the enhanced expression of RhoA and ROCK1 at the protein level (Fig. 3B,C). Y‐27632 completely canceled the enhanced expression of ROCK1 in HSCs with the exposure to 50 mmHg pressure (Fig. 3), although Y‐27632 also significantly decreased the expression of ROCK2 (Fig. 3).

**Fig. 4:**
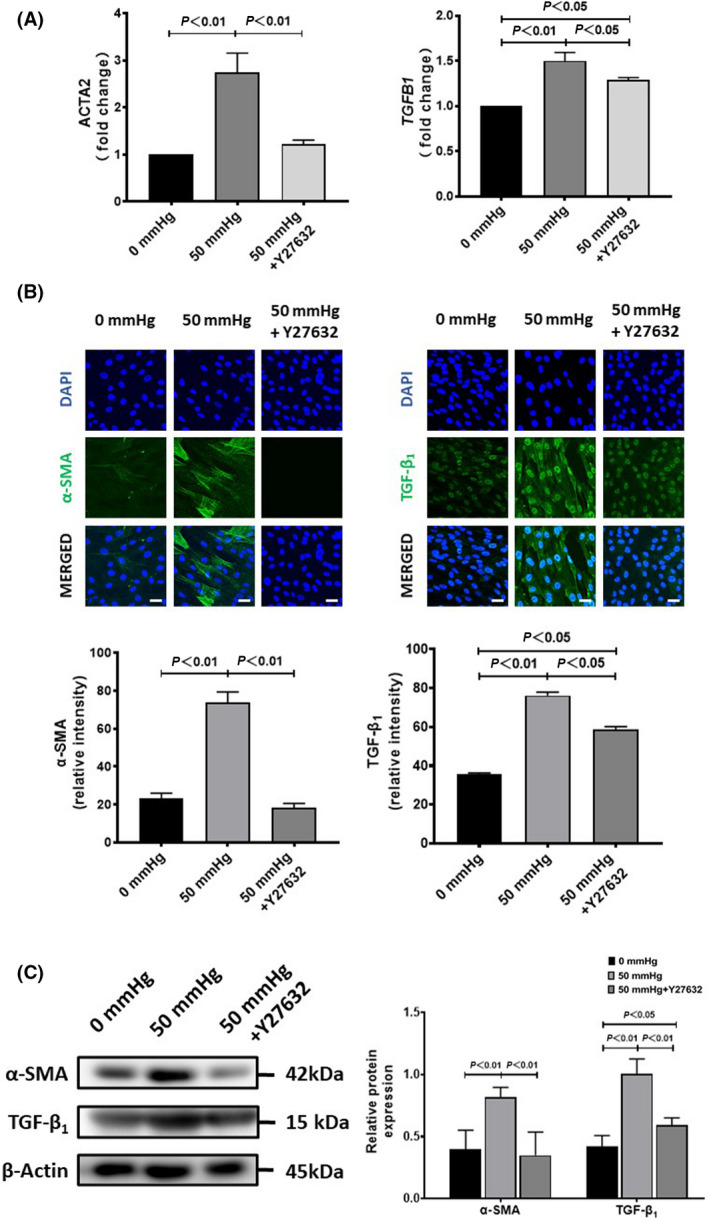
The expression of α‐SMA and TGF‐β_1_ in human hepatic stellate cells (HSCs) after exposure to 50 mmHg pressure for 24 h with or without the addition of Y‐27632 in medium. (A) Quantitative RT‐qPCR data on the fold change of mRNA expression levels (vs. 0 mmHg). (B) Representative images (upper) and semiquantitative data on the immunofluorescence staining intensity. Scale bars = 30 μm. (C) The protein expression by western blot analysis is also shown. Data are represented as mean ± SD from three independent experiments. *P* values were analyzed by one‐way ANOVA.

**Fig. 5:**
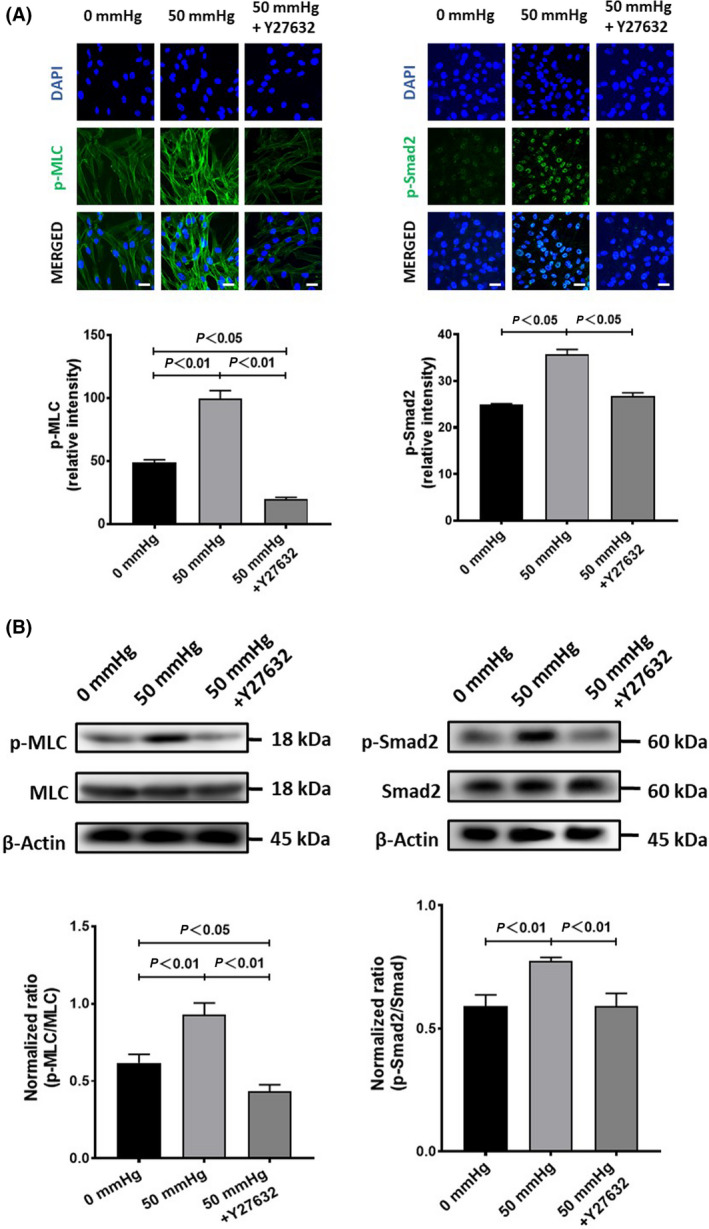
The phosphorylation of MLC and Smad2 in human hepatic stellate cells (HSCs) after exposure to 50 mmHg pressure for 24 h with or without the addition of Y‐27632 in medium. (A) Representative images (upper) and semiquantitative data on the immunofluorescence staining intensity. Scale bars = 30 μm. (B) The protein expression by western blot analysis is also shown. Data are represented as mean ± SD from three independent experiments. *P* values were analyzed by one‐way ANOVA.

Moreover, the exposure of HSCs to 50 mmHg pressure for 24 h significantly increased the expression of α‐SMA and TGF‐β_1_ at either the mRNA or protein levels (Fig. 4A,B,C). The blockade of RhoA/ROCK signaling by Y‐27632 completely canceled the enhanced expression of α‐SMA, and partially canceled the enhanced expression of TGF‐β_1_ in HSCs with the exposure to 50 mmHg pressure (Fig. 4A,B,C). Consistent with the upregulation of RhoA and ROCK1, the phosphorylated MLC was significantly upregulated in HSCs exposed to 50 mmHg pressure for 24 h (Fig. 5A,B), but was effectively abolished by Y‐27632. Otherwise, the phosphorylated Smad2 in HSCs was also upregulated by 50 mmHg pressure but was cancelled by Y‐27632 (Fig. 5A,B). These data suggested that RhoA/ROCK signaling was involved in the acquisition of the profibrotic property of HSCs with hydrostatic pressure exposure.

## Discussion

In this study we tried to investigate the probable role and relevant mechanism of hydrostatic pressure on the biological property of HSCs. By mimicking the elevated hydrostatic pressure in diseased livers, we exposed *ex vivo* primary human HSCs to 20 and 50 mmHg pressure using a commercial device. We found that the exposure of HSCs to 50 mmHg pressure for 24 h clearly induced the acquisition of the profibrotic property. We demonstrated that the RhoA/ROCK signaling involved in the hydrostatic pressure‐induced change on the biological property of HSCs.

Dynamic alternations of biomechanical forces, especially the hydrostatic pressure and stiffness, are commonly observed in diseased livers [10]. Although biomechanical forces have been well known to regulate a variety of cellular properties and activities, the precise role and the relevant molecular/cellular mechanisms of biomechanical forces in the initiation and progression of liver diseases are still poorly understood. The complex changes of tissue factors, including various biomechanical forces within the microenvironment of liver under different pathological conditions largely limited the approach of *in vivo* evaluations. Therefore, an *ex vivo* approach has been recently applied to investigate the potential effect and molecular mechanisms of biomechanical forces on the biological properties of cells, including HSCs [19].

Elevated hydrostatic pressure and excessive accumulation of ECM are almost uniformly observed in diseased livers. The excessive accumulation of ECM in liver results in not only the initiation and progression of fibrogenesis [14, 20], but also the alteration of stiffness, a type of biomechanical force. Moreover, the excessive accumulation of ECM may induce the elevation of hydrostatic pressure. However,it the causal relationship between the elevated hydrostatic pressure and liver fibrosis has been poorly understood. Considering that the interstitial fluid hydrostatic pressure in liver can be quickly elevated due to fluid trapping in the acute phase of different pathological disorders, it will be reasonable to speculate that an elevated hydrostatic pressure may induce the activation of HSCs to initiate the accumulation of ECM and accelerate liver fibrosis.

To simply examine our speculation by the *ex vivo* approach, we pressurized human HSCs using a pneumatic pressurizing system to mimic the elevated hydrostatic pressure *in vivo*. As hepatic venous pressure can be elevated to 30 mmHg in patients with diseased liver [9], we decided to test first by exposing HSCs to 20 and 50 mmHg. The PCR array data showed that the expression of *ACTA2*, *COL1A1,* and *COL1A2* were upregulated in HSCs exposed to either 20 mmHg or 50 mmHg pressure, suggesting the activation and acquisition of fibrotic properties. However, the gene expression changes from the PCR array data were more clearly detected in HSCs exposed to 50 mmHg pressure compared to 20 mmHg. A previous study has reported on the increased proliferation rate of activated HSCs [21], but the cell survival/proliferation of HSCs was not significantly changed by 24 h of exposure to 50 mmHg pressure.

Considering that the clinical hepatic venous pressure in diseased livers can be elevated to ~ 30 mmHg [9], we selected 50 mmHg in most of the experiments for understanding the relevant molecular mechanisms. We only focused on RhoA/ROCK signaling in this study because RhoA/ROCK signaling has been well known to play central role in the conversion of biomechanics into a defined biochemical output by regulating cytoskeletal properties [4, 5]. Mechanical forces can be sensitized by mechanosensors such as integrins, which directly activates RhoA/ROCK signaling through bridging proteins [22]. As one of the major mechanotransduction pathways, the activation of RhoA/ROCK signaling can directly or indirectly change the expression of TGF‐β and α‐SMA, which thereby induces fibrogenesis [23, 24].

An increased expression of RhoA/ROCK has also been observed in the HSCs and hepatocytes after biomechanical stimulations [25, 26]. In addition, F‐actin cytoskeleton reorganization has been reported to be related to the activation of HSC [27]. Our *ex vivo* experimental data indicated that 50 mmHg hydrostatic pressure clearly induced the acquisition of the profibrotic properties of HSCs through RhoA/ROCK signaling. Interestingly, our data showed that ROCK1, but not ROCK2, mediated the activation of HSCs, which may due to the ubiquitous expression of ROCK1 in liver [28].

As one of the most potent fibrogenic cytokines [29, 30], TGF‐β_1_ is generally considered to promote and maintain the activation of HSCs through the canonical Smad pathway [30, 31, 32]. Besides, the upregulation of TGF‐β_1_ has also been found in lung myofibroblasts responding to biomechanical stimulation [33]. Agreeing well with these previous studies, our data showed that the exposure to 50 mmHg pressure extensively upregulated the TGF‐β superfamily genes, including TGF‐β_1_ and Smad2 in HSCs. We further confirmed that the ROCK inhibitor Y‐27632 significantly, but not completely, attenuated the enhanced expression of TGF‐β_1_, suggesting that the RhoA/ROCK signaling was involved, at least partially, in the mechanotransduction of HSCs in response to hydrostatic pressure stimulation. As the enhanced expressions of TGF‐β_1_ were not completely canceled by Y‐27632, other molecular signaling may also involve in the hydrostatic pressure‐induced activation of HSCs.

HSCs, as the primary effector cells, acquiring a profibrotic phenotype is a key link in liver fibrosis by orchestrating the deposition of ECM in normal and fibrotic liver [34]. As a proof‐of‐concept study, we used primary human HSCs, rather than an established cell line for experiments. Although the sensitivity and responsibility may be varied depending on the cell quality and culture conditions, we believe that the main findings and essential conclusion of this study will not be largely changed by using other primary HSCs or established cell lines for experiments.

In conclusion, data from our *ex vivo* experiments clearly demonstrated that hydrostatic pressure activated HSCs to acquire a profibrotic property, likely through the RhoA/ROCK signaling pathway. Uncovering the biomechanical signaling pathway on the activation of HSCs will be helpful to develop novel molecular targeting therapy for liver diseases.

## Conflict of interest

The authors declare no conflicts of interest.

## Author contributions

WG and T‐SL conceived and designed the research. ZH and MOK performed experiments. ZH analyzed the data. ZH prepared the figures. WG and T‐SL edited and revised the article. WG and T‐SL approved the final version of the article.

## Supporting information


**Table S1.** Primers used for quantitative RT‐PCR.
**Table S2.** Details of primary antibodies.
**Table S3.** Detail of secondary antibodies.
**Table S4.** Human fibrosis RT2 Profiler™ PCR array data on the fold changes (vs. 0 mmHg) of genes in hepatic stellate cells after 24 h of exposure to 20 or 50 mmHg pressure.

## Data Availability

The data that support the findings of this study are available from the corresponding author (litaoshe@nagasaki-u.ac.jp) upon reasonable request.
