# Supplementary material for: Hydrostatic pressure induces profibrotic properties in hepatic stellate cells via the RhoA/ROCK signaling pathway
Source: FEBS Open Bio. 2022 Apr 15;12(6):1230–40. doi: 10.1002/2211-5463.13405 (PMC9157409; doi:10.1002/2211-5463.13405)
Supplement: Supplementary file 1 — Table S1. Primers used for quantitative RT‐PCR. Table S2. Details of primary antibodies. Table S3. Detail of secondary antibodies. Table S4. Human fibrosis RT2 Profiler™ PCR array data on the fold changes (vs. 0 mmHg) of genes in hepatic stellate cells after 24 h of exposure to 20 or 50 mmHg pressure. [file FEB4-12-1230-s001.docx]

**Supplementary Table**

**Supplementary Table 1**. Primers used for quantitative RT-PCR

| Genes | Forward (5'→3') | Reverse (5'→3') |
| --- | --- | --- |
| *RHOA* | CAGAAAAGGGACCCCAGAA | GCAGCTGCTCTCGTAGCCATTTC |
| *ROCK1* | AACATGCTGCTGGATAAATCTGG | AGGAAGGCATGGTACGATGTGATACA |
| *ROCK2* | TCAGAGGT CTACAGATGAAGGC | CCAGGGGCTATTGGCAAAGG |
| *ACTA2* | GTGACGAAGCACAGAGCAAA | CTTTTCCATGTCGTCCCAGT |
| *TGFB1* | CTCTCCGACCTGCCACAGA | AACCTAGATGGGCGCGATCT |
| *GAPDH* | GACTCATGACCACAGTCCATGC | AGAGGCAGGGATGATGTTCTG |

**Supplementary Table 2**. Details of primary antibodies

| antibody | manufacturer | Catalog number |
| --- | --- | --- |
| Mouse Anti-RhoA | SantaCruz | SC-418 |
| Rabbit Anti-ROCK1 | Abcam | ab97592 |
| Rabbit Anti-ROCK2 | Abcam | ab71598 |
| Rabbit Anti-Collagen I | Abcam | ab34710 |
| Rabbit Anti-TGF-beta1 | Abcam | ab92486 |
| Rabbit Anti-α-Smooth muscle actin | CST | 19245 |
| Rabbit Anti-Phospho-myosin light chain 2 | CST | 3674 |
| Rabbit Anti-Myosin light chain 2 | CST | 3672 |
| Rabbit Anti-phospho-Smad2 | CST | 8828 |
| Rabbit Anti- -Smad2 | CST | 5339 |
| Rabbit Anti-β-Actin | CST | 8457 |

**Supplementary Table 3**. Detail of secondary antibodies

| antibody | manufacturer | Catalog number | |
| --- | --- | --- | --- |
| Goat Anti-rabbit (Alexa Fluor 488) | Invitrogen | A32731 |  |
| Goat Anti-mouse (Alexa Fluor 488) | Invitrogen | A11029 |  |

**Supplementary Table 4.** Human fibrosis RT2 Profiler™ PCR array data on the fold changes (vs. 0 mmHg) of genes in hepatic stellate cells after 24 hours of exposure to 20 or 50 mmHg pressure.

| **Genes** | **Fold change** | |  | **Genes** | **Fold change** | |
| --- | --- | --- | --- | --- | --- | --- |
|  | **20 mmHg** | **50 mmHg** |  |  | **20 mmHg** | **50 mmHg** |
| *MMP1* | -1.23 | 9.29 |  | *ITGB6* | -2.05 | -1.97 |
| *MMP3* | 1.57 | 4.71 |  | *INHBE* | 2.89 | 6.47 |
| *MMP14* | -2.47 | 3.40 |  | *GREM1* | 1.62 | 5.22 |
| *TIMP4* | -1.90 | 2.86 |  | *TGFB2* | 1.19 | 2.24 |
| *MMP2* | 1.20 | 2.39 |  | *TGIF1* | 1.54 | 2.21 |
| *TIMP2* | -1.76 | 2.09 |  | *SMAD7* | -3.52 | 1.95 |
| *PLAT* | -7.82 | 2.01 |  | *TGFB1* | 1.68 | 1.89 |
| *SERPINE1* | -3.43 | 1.90 |  | *THBS2* | -6.39 | 1.76 |
| *TIMP3* | -1.16 | 1.72 |  | *SMAD2* | -1.86 | 1.58 |
| *LOX* | 1.04 | 1.62 |  | *TGFBR1* | 1.18 | 1.56 |
| *SERPINH1* | 1.27 | 1.30 |  | *ENG* | -4.46 | 1.50 |
| *TIMP1* | -1.63 | 1.24 |  | *THBS1* | -6.09 | 1.10 |
| *PLAU* | -2.75 | 1.02 |  | *TGFBR2* | -2.67 | 1.48 |
| *MMP13* | 1.46 | -1.07 |  | *SMAD4* | -2.18 | 1.33 |
| *MMP9* | 1.37 | -1.29 |  | *LTBP1* | -1.43 | -1.21 |
| *PLG* | 1.46 | -1.85 |  | *TGFB3* | -2.26 | -1.60 |
| *MMP8* | 1.27 | -2.08 |  | *SMAD6* | -2.39 | -2.58 |
| *CAV1* | -1.86 | -1.87 |  | *IFNG* | -1.66 | -4.10 |
| *AGT* | 5.16 | 6.11 |  | *SMAD3* | -4.02 | -7.44 |
| *VEGFA* | 2.59 | 6.57 |  | *CEBPB* | -1.06 | 3.75 |
| *CTGF* | 1.04 | 2.08 |  | *JUN* | -12.35 | 1.69 |
| *PDGFB* | -1.19 | 1.89 |  | *STAT1* | -1.54 | 1.58 |
| *PDGFA* | -1.10 | 1.45 |  | *SP1* | -2.98 | 1.36 |
| *EDN1* | -2.58 | 1.12 |  | *STAT6* | -2.71 | 1.26 |
| *DCN* | 1.09 | -1.07 |  | *MYC* | -1.50 | 1.02 |
| *HGF* | -7.19 | -3.11 |  | *NFKB1* | -2.07 | -1.09 |
| *EGF* | 2.03 | -4.48 |  | *IL13RA2* | 2.45 | 2.86 |
| *SNAI1* | -3.47 | 1.28 |  | *TNF* | 1.46 | 1.98 |
| *AKT1* | -4.19 | 1.06 |  | *IL1A* | 1.14 | 1.35 |
| *COL1A2* | 1.58 | 1.59 |  | *IL1B* | -1.58 | 1.13 |
| *COL3A1* | 1.48 | 1.86 |  | *ILK* | -1.66 | 1.04 |
| *SERPINA1* | -3.43 | -1.11 |  | *IL13* | -3.38 | -1.08 |
| *BMP7* | -1.70 | -1.70 |  | *CCL3* | 1.46 | -1.14 |
| *ITGB5* | -7.19 | 2.72 |  | *CXCR4* | -1.47 | -1.14 |
| *ITGA3* | -1.55 | 2.14 |  | *CCR2* | 1.92 | -1.18 |
| *ITGA2* | -2.36 | 1.82 |  | *IL4* | -1.00 | -2.47 |
| *ITGAV* | -1.60 | 1.68 |  | *CCL2* | -1.86 | -2.50 |
| *ITGB1* | -1.34 | 1.51 |  | *CCL11* | -2.05 | -3.33 |
| *ITGA1* | -1.15 | 1.41 |  | *IL10* | -2.60 | -5.22 |
| *ITGB3* | -2.90 | -1.28 |  | *IL5* | -3.43 | -8.03 |
| *ITGB8* | -1.08 | -1.51 |  | *ACTA2* | 1.46 | 1.65 |
